# Improving Blood Pressure Control in Patients with Diabetes Mellitus and High Cardiovascular Risk

**DOI:** 10.4061/2010/490769

**Published:** 2011-01-10

**Authors:** Henry L. Elliott, Suzanne M. Lloyd, Ian Ford, Peter A. Meredith

**Affiliations:** ^1^Institute of Pharmaceutical and Biomedical Sciences, University of Strathclyde, Glasgow G1 1XQ, UK; ^2^Robertson Centre for Biostatistics, University of Glasgow, Boyd Orr Building, Glasgow G12 8QQ, UK; ^3^Division of Cardiovascular and Medical Sciences, University of Glasgow, Western Infirmary, Glasgow G11 6NT, UK

## Abstract

Patients with diabetes mellitus and symptomatic coronary artery disease are also likely to be hypertensive and, overall, are at very high cardiovascular (CV) risk. This paper reports the findings of a posthoc analysis of the 1113 patients with diabetes mellitus in the ACTION trial: ACTION itself showed that outcomes in patients with stable angina and hypertension were significantly improved when a long-acting calcium channel blocking drug (nifedipine GITS) was added to their treatment regimens. This further analysis of the ACTION database in those patients with diabetes has identified a number of practical therapeutic issues which are still relevant because of potential outcome benefits, particularly in relation to BP control. For example, despite background CV treatment and, specifically, despite the widespread use of ACE Inhibitor drugs, the addition of nifedipine GITS was associated with significant benefits: improvement in BP control by an average of 6/3 mmHg and significant improvements in outcome. In summary, this retrospective analysis has identified that the addition of nifedipine GITS resulted in improved BP control and significant outcome benefits in patients with diabetes who were at high CV risk. There is evidence to suggest that these findings are of direct relevance to current therapeutic practice.

## 1. Introduction

The optimal management of patients with type 2 diabetes mellitus requires a multiplicity of drug treatments: not only for glycaemic control but also for nephroprotection and for reducing cardiovascular (CV) risk. Since CV disease, particularly coronary artery disease (CAD), accounts for around 60% of deaths in people with type 2 diabetes mellitus, “cardioprotective” drugs are obviously of fundamental importance [1, 2]. Furthermore, since many of these patients with diabetes also have hypertension and since there is good evidence that “tight” blood pressure (BP) control significantly reduces CV morbidity and mortality in diabetic, hypertensive patients (usually by means of combination drug treatment), optimal antihypertensive treatment is central to the overall therapeutic strategy [3, 4]. Although it is recommended that the antihypertensive treatment regimen includes drugs which block the renin-angiotensin system (RAS blockade) (mainly because of the evidence suggestive of nephroprotection) [5–7], it is important to remember that RAS blockade has no direct antianginal activity. Thus, there is not yet agreement on the “best practice” antihypertensive/antianginal treatment combination for patients with diabetes, hypertension, and symptomatic CAD [8–12].

The ACTION trial (published in 2004) established that the addition of nifedipine (in its long-acting GITS formulation) improved the prognosis of patients with chronic stable angina, particularly in those with concomitant hypertension [13, 14]. This further analysis specifically addresses practical treatment issues in more than 1000 patients with high CV risk which was attributable to the combination of established CAD, diabetes, and hypertension. Attention is directly focused on the role of improved BP control by means of the calcium channel blocking drug (CCB), nifedipine, which has well-recognised pharmacological properties known to lead to BP reduction, symptomatic improvement in angina, and amelioration of underlying myocardial ischaemia [15, 16].

## 2. Patients and Methods

The design, selection criteria, methods, and main results of the ACTION trial have been published in detail previously [13, 14, 17]. In brief, 7655 patients with symptomatic, stable angina pectoris were randomized to receive either nifedipine GITS (*n* = 3825) or matching placebo (*n* = 3840). In addition to angina, patients had to have either a history of myocardial infarction (MI), or proven angiographic coronary artery disease, or a positive exercise test or perfusion defect. The left ventricular ejection fraction had to be at least 40%. The definition of diabetes mellitus (DM) was simplistically based upon the “clinical history” according to the ACTION study investigators with the caveat that patients with unstable type 1 DM were ineligible [17]. The starting dose of nifedipine GITS or placebo was 30 mg once daily, increasing to 60 mg once daily within 6 weeks. It is important to note that these treatments were in addition to “best practice” CV drug therapy (at the time), with a follow-up period of 4 to 5 years.

This further analysis has explored a number of aspects of the effectiveness of treatment with nifedipine GITS in patients with the combination of chronic stable angina and diabetes mellitus.

## 3. Statistical Methods

Five composite endpoints were investigated: these were all prespecified in the ACTION protocol and investigated as part of the main study analysis. These endpoints were defined as: *the primary efficacy endpoint* (all-cause mortality, MI, refractory angina requiring coronary angiography, new overt heart failure requiring hospitalization, and peripheral revascularisation); *the primary safety endpoint* (all-cause mortality, MI, and debilitating stroke); *any cardiovascular event* (primary endpoint for efficacy minus noncardiovascular death); *any death, cardiovascular event, or procedure* (i.e., the primary endpoint for efficacy plus coronary angiography, percutaneous coronary intervention (PCI), and coronary bypass surgery (CABG)); and *any vascular event or procedure* (i.e., the primary endpoint for efficacy minus noncardiovascular death and new overt heart failure plus PCI and CABG.

 Patients diagnosed with diabetes mellitus at baseline were compared to the nondiabetic patients in terms of a number of baseline characteristics using two-sample *t*-tests and Fisher's exact test for continuous and categorical variables, respectively. Using Fisher's exact test, concomitant medications were compared for the two patient groups; diabetics and nondiabetics on the same treatment were also compared. To illustrate the antihypertensive effect of nifedipine GITS, the numbers of patients (%) achieving the arbitrary target levels of either 140/90 or 130/80 mmHg were summarised by year, diabetic status, and treatment group and are presented in the form of bar charts. The safety of nifedipine was further explored by plotting the mean changes in glucose and creatinine over time by diabetic status and treatment group.

Hazard ratios and 95% confidence intervals were obtained in the analyses of the five composite endpoints from Cox proportional hazards models for the treatment effect within each of the diabetic subgroups; interactions between diabetes and treatment were also investigated. Subgroup analyses of patients receiving RAS blockade are also presented. All analyses were carried out on the intention-to-treat principle using SAS v 9.1.

## 4. Results

**Figure 1:**
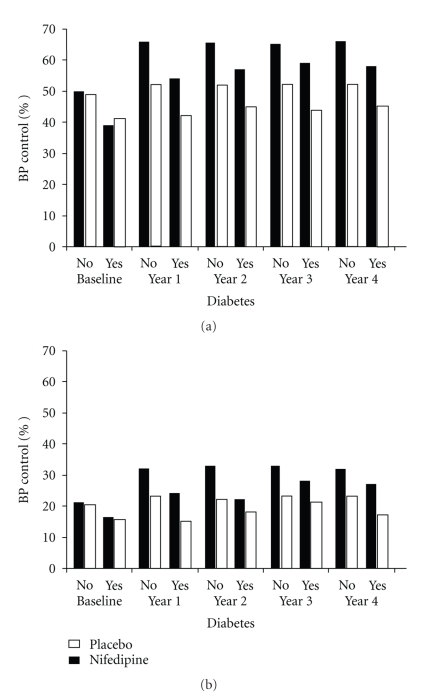
Patients (%) in different subgroups achieving BP Control at <140/90 (a) and <130/80 mmHg (b).

Of 7665 patients started on study medication, 1113 (14.5%) were categorised with diabetes mellitus at baseline: the great majority, approximately 85%, were classified as patients with type 2 diabetes. The baseline clinical characteristics are summarized by diabetic status in Table 1. The patient cohorts—those with diabetes and those without diabetes—were similar in terms of age, gender, and a number of clinical features. However, there were some differences, for example, in the patients without diabetes there were more current cigarette smokers and more patients with total cholesterol above 5 mmol/l. These differences obviously would affect CV risk in the group without diabetes but the adverse impact of diabetes itself outweighs these small percentage differences and this is reflected in the rates for events and procedures shown in Table 2, whereby the patients with diabetes invariably have higher rates for all-cause death, major events, vascular procedures, and so forth. With particular respect to hypertension there were obvious, statistically significant and clinically relevant differences: for example, 56% of the patients with diabetes were already receiving drug treatment for hypertension but 60% remained uncontrolled with BP above the conventional target of 140/90 mmHg (the mean BP was 141/80 mmHg). The corresponding values in the patients without diabetes were significantly different with 39% receiving antihypertensive drug treatment, with 51% remaining uncontrolled (>140/90) and a mean BP of 137/80 mmHg. The attainment of different levels of BP control is illustrated in Figure 1. After 4-year of nifedipine treatment, 58% of the patients with diabetes had achieved BP <140/90 compared to 45% in those treated with placebo: similar differences were apparent for the patients without diabetes at, respectively, 66 and 52% Figure 1(a). Tighter BP control to <130/80 mmHg was achieved in 27% of the patients with diabetes treated with nifedipine compared to 17% of patients on placebo; again, similar proportions at, respectively, 32 and 23% were found in the nondiabetic patients Figure 1(b).

 Concomitant medications are summarised in Table 3. Throughout the study there were small but significant differences between the treatment groups. Patients with diabetes were more likely to receive drugs acting on the renin-angiotensin system (RAS blockade), either an ACE-inhibitor or an angiotensin receptor blocker, either at baseline or at some time during the study. For example, 34% of the patients with diabetes randomised to nifedipine were receiving RAS blockade at baseline compared to 20% of the nondiabetic patients (*P* < .0001) and correspondingly 65% compared to 40% at any time during the study (*P* < .0001). Additionally, both patients with and without diabetes receiving nifedipine were significantly less likely to receive additional antihypertensive treatment during the study. For example, in the cohort of patients with diabetes, RAS blockade was incorporated at some time during the study in 72% of patients randomized to placebo (65% in the nifedipine group; *P* < .02) and diuretic was added in 53% of patients (46% in the nifedipine group; *P* < .05).

**Figure 2:**
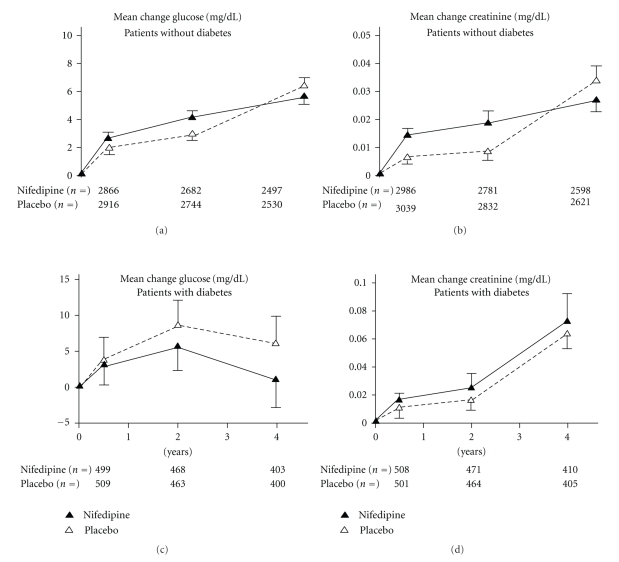
Laboratory values: creatinine (b,d) and glucose (a,c) in patients with diabetes (c,d) and without diabetes (a,b).

 The incidental effects of 4 years treatment with nifedipine on glucose control and on renal function (serum creatinine) are illustrated in Figure 2. There were slight increases over time in serum creatinine: these increases were seen in both treatment groups and for both diabetic and nondiabetic patients. Similar findings were seen for plasma glucose with a trend for smaller increases in the nifedipine treatment group after 6 months. None of the differences, for either creatinine or glucose, attained statistical significance.

**Figure 3:**
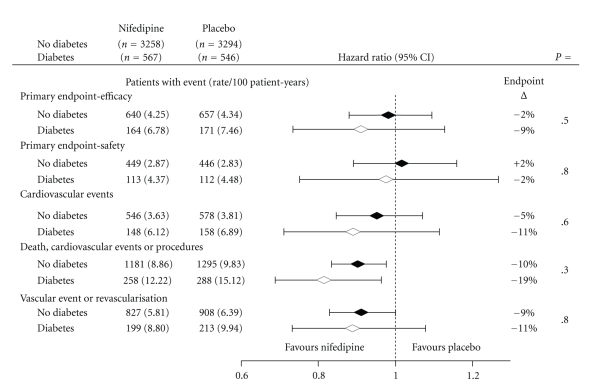
Major outcomes in diabetic and nondiabetic patients.

 The treatment effects are summarised by diabetic status for each of the major endpoints in Figure 3. Although there were no statistically significant differences, there was an overall pattern whereby the magnitude of the benefits associated with nifedipine treatment was at least as great in the diabetic cohort. For example, the composite endpoint of CV events, death and procedures showed a significant reduction by 10% in the nondiabetic population and a significant reduction of 19% in the diabetic population. The rates of major individual end-points and procedures are shown in Table 2: there were no statistically significant differences. The all-cause death rate was nonsignificantly higher but the benefits attributable to nifedipine GITS reflected fewer major clinical events, fewer interventions, fewer investigations, and fewer hospitalizations for refractory angina.

**Figure 4:**
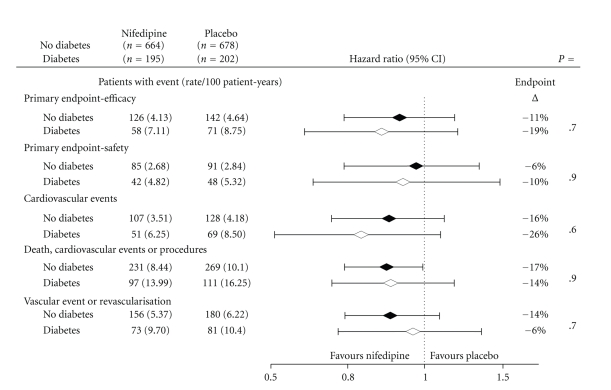
Major outcomes in patients receiving combination treatment with RAS blockade and Nifedipine GITS.

The subgroup analysis of those patients with diabetes receiving RAS blockade showed a similar pattern of benefit for those patients receiving nifedipine GITS *in addition* to RAS blockade (Figure 4).

## 5. Discussion

The combination of hypertension and coronary artery disease occurs more often in patients with type 2 diabetes than in matched controls [2]. This analysis of the subgroup of patients with diabetes mellitus within the ACTION study population focuses attention on practical therapeutic issues in such high-risk patients. The weaknesses of such retrospective, subgroup analyses (with relatively modest statistical power) are readily acknowledged but the findings and resultant conclusions remain directly relevant to current clinical practice. 

The ACTION study involved adding either nifedipine GITS or placebo to “best practice” CV treatment and it was disappointing to find that, at baseline, 88% of the patients with diabetes mellitus (and 87% of the patients without diabetes) had treatable CV risk factors over and above their underlying CAD. It is particularly disappointing that “best practice treatment” in patients with CAD and diabetes “tolerated” BP levels above 140/90 mmHg in 60% and total cholesterol above 5 mmol/l also in 60% of patients. Whilst awareness of the importance of risk factor management and the relevance of “aggressive” treatment targets has considerably increased in recent years, these percentages are not markedly different from those reported from USA in 2009 [18]. In this American survey, 54.5% of the patients with diabetes had inadequate BP control (>130/80 mmHg), and 53.5% had suboptimal cholesterol levels (total cholesterol >5 mmol/l). This survey additionally noted that the triple combination of glycaemic control, BP control and lipid control was achieved in only 12.2% of patients: therefore, 87.8% had at least one uncontrolled CV risk factor [18]. Thus, although risk factor management has progressively and significantly improved in recent years, there remain shortcomings in the management of patients with diabetes and high CV risk: these are precisely the shortcomings identified in this posthoc ACTION analysis. 

Focusing on antihypertensive drug treatment, the addition of nifedipine GITS in ACTION resulted in a significant improvement in BP control through a mean BP reduction of 6/3 mmHg. This is both a statistically significant improvement and a clinically relevant improvement. There is clear evidence that an improvement of this magnitude in BP control will significantly improve prognosis in patients with diabetes [3, 4]. However, closer scrutiny of the BP data in the cohort of patients with diabetes reveals that BP control at a target level of less than 140/90 was attained in less than 50% of patients (and a target of less than 130/80 mmHg in only 27%, albeit compared to 16% at baseline). Some possible practical explanations for these disappointing results are obviously that diabetic hypertension is particularly difficult to control and/or that the importance of BP control was undervalued (and the CV risk underestimated) by the participating physicians at that time. Irrespective of the quality and nature of the background treatment, the addition of nifedipine GITS in ACTION was associated not only with improved BP control but also with significant outcome benefits in the cohort of patients with diabetes. Clearly, it is possible that the outcome benefits were simply attributable to the improvement in BP control and that the addition of a different long-acting CCB, such as amlodipine or felodipine, might have achieved similar success. However, although amlodipine has similar pharmacological activity and a similar therapeutic profile [19], there are no corresponding data in patients with the clinical profile of diabetes, hypertension and symptomatic, stable CAD. Similarly, there is no directly comparable evidence with felodipine, but the results of the HOT study provide supportive evidence of the benefits of modest improvements in BP control in high-risk patients with diabetes [3].

Another consideration relates to the use of RAS blockade. The recently revised European Hypertension guidelines continue to recommend RAS blockade as a first-line treatment for hypertension in patients with diabetes, but this reflects the evidence relating to proteinuria and nephroprotection and not directly to “cardioprotection” [20]. Thus, in the patients with diabetes in ACTION, “reliance” may have been placed on the putative cardioprotective properties of drugs blocking the renin-angiotensin system (RAS blockade) rather than the attainment of improved BP control [21]. In this regard, between 65 and 72% of the patient population with diabetes in ACTION received these drugs at some time during the study. However, RAS blocking drugs have no direct antianginal/antiischaemic properties (in contrast to nifedipine [22]). It is interesting to note that favourable results (i.e., benefits of greater magnitude) were obtained in the whole ACTION population in those patients receiving nifedipine GITS *in addition* to RAS blockade [23]. 

In conclusion, although this posthoc analysis reflects clinical practice from almost a decade ago, the most recent survey from the USA identifies that there remain problems with optimal CV risk factor management in patients with diabetes and high CV risk. Firstly, in such high-risk patients with diabetes and increased levels of BP (and cholesterol), targets are difficult to achieve. However, significant improvements are possible: for example, improved BP control by the simple means of additional drug treatment (in this case, a long-acting CCB). Secondly, in treatment specific terms, the evidence suggests that RAS blockade alone (with either an ACE inhibitor or an angiotensin receptor blocker) may not provide sufficient BP control or “cardioprotection”. In such patients, with high CV risk because of diabetes, hypertension and symptomatic, chronic stable angina, there were statistically significant benefits via increased rates of BP control and improved outcomes (and fewer vascular procedures) when a different type of drug (nifedipine GITS) with proven antihypertensive, antianginal and antiischaemic properties was added to the multi-drug treatment regimen.

## Figures and Tables

**Table 1:** Summary of patient characteristics (at baseline).

	Without-diabetes (*n* = 6552)	With-diabetes (*n* = 1113)	*P*-value
*Basic Demography*			
Age (years)	63.4 (9.4)	63.9 (8.9)	.082
Males	5224 (80%)	860 (77%)	.065
Body Mass Index (kg/m^2^)	27.3 (3.7)	28.7 (4.2)	<.0001

*Clinical Features*			
History of MI	3326 (51%)	572 (51%)	.721
History of PTCA	1720 (26%)	296 (27%)	.825
History of CABG	1486 (23%)	303 (27%)	.001
Claudication	451 (7%)	174 (16%)	<.0001
Transient ischaemic attack	247 (4%)	55 (5%)	.067
Stroke	139 (2%)	31 (3%)	.185

*Drug Treatment*			
(i) for hypertension	2577 (39%)	621 (56%)	<.0001
(ii) for hyperlipidaemia	4446 (68%)	754 (68%)	.945

*Laboratory Variables*			
Glucose (mmol/l)	4.46 (1.17)	9.71 (3.89)	<.0001
Creatinine (umol/l)	97.2 (17.7)	97.2 (17.7)	.890

*Cardiovascular Variables*			
Pulse Rate (beats/min)	63.8 (10.1)	67.1 (10.7)	<.0001
Systolic BP (mmHg)	136.9 (18.8)	140.7 (18.0)	<.0001
Diastolic BP (mmHg)	79.9 (9.5)	79.8 (9.4)	.719

*Risk Factors*			
Current Smoker	1204 (18%)	152 (14%)	.0001
Total cholesterol ≥5.0 mmol/L	4167 (65%)	648 (60%)	.002
Body Mass Index ≥30.0 kg/m^2^	1368 (21%)	376 (34%)	<.0001
Blood pressure ≥140/90 mmHg	3309 (51%)	668 (60%)	<.0001
Any of the above	5669 (87%)	984 (88%)	.094

Data are presented as number of patients (%) or mean (SD).

**Table 2:** Rates (per 100 patient years) for individual end-points.

End-point	Without diabetes			With diabetes			*P-value
	Nifedipine	Placebo	Hazard ratio (95% C. I.)	Nifedipine	Placebo	Hazard ratio (95% C. I.)	
All-cause death	1.53	1.43	1.07 (0.89, 1.28)	2.27	2.15	1.05 (0.74, 1.51)	.942
Myocardial Infarction	1.36	1.25	1.09 (0.9, 1.32)	2.02	2.28	0.89 (0.61, 1.29)	.326
Heart Failure	0.37	0.51	0.72 (0.52, 1.01)	1.01	1.52	0.67 (0.41, 1.49)	.771
Stroke	0.33	0.49	0.67 (0.47, 0.94)	0.9	0.73	1.22 (0.67, 2.23)	.086
Coronary Artery Bypass Grafting	1.46	1.92	0.76 (0.64, 0.9)	2.61	2.92	0.9 (0.64, 1.25)	.395
Percutaneous Coronary Intervention	2.08	2.21	0.94 (0.81, 1.1)	2.57	3.11	0.83 (0.59, 1.15)	.482
Peripheral Revascularisation	0.72	0.56	1.27 (0.96, 1.67)	1.21	1.05	1.16 (0.7, 1.94)	.749
Refractory Angina	0.76	0.86	0.89 (0.7, 1.14)	1.09	1.50	0.73 (0.45, 1.18)	.47
Coronary Angiography	5.22	6.29	0.83 (0.75, 0.92)	6.93	9.28	0.75 (0.61, 0.93)	.384

*P-value for diabetes status/treatment interaction term.

**Table 3:** Further details of cardiovascular drugs and diabetes treatments.

	Without diabetes			With diabetes			*P*-value for Nifedipine	*P*-value for Placebo comparison
	Nifedipine	Placebo	*P*-value	Nifedipine	Placebo	*P*-value	comparison	
Baseline								

Cardiovascular Drugs								
Beta-Blocker	2583 (79%)	2632 (80%)	—	450 (79%)	434 (80%)	—	1.000	.818
ACE-I or ARB	664 (20%)	678 (21%)	—	195 (34%)	202 (37%)	—	<.0001	<.0001
Diuretic	350 (11%)	359 (11%)	—	82 (15%)	88 (16%)	—	.012	<.0001
Any Blood								
Glucose	—	—	—	434 (77%)	419 (77%)	—	—	—
Lowering Rx								
Insulin	—	—	—	88 (16%)	96 (18%)	—	—	—
Metformin	—	—	—	177 (31%)	159 (29%)	—	—	—
Sulfonylureas	—	—	—	302 (53%)	282 (52%)	—	—	—

At any time								

Cardiovascular Drugs								
Beta-Blocker	2868 (88%)	2943 (89%)	.094	506 (89%)	495 (91%)	.486	.438	.406
ACE-I or ARB	1298 (40%)	1583 (48%)	<.001	371 (65%)	393 (72%)	.020	<.0001	<.0001
Diuretic	1130 (35%)	1256 (38%)	.004	261 (46%)	287 (53%)	.031	<.0001	<.0001
